# Connecting Aortic Stiffness to Vascular Contraction: Does Sex Matter?

**DOI:** 10.3390/ijms231911314

**Published:** 2022-09-25

**Authors:** Amanda A. de Oliveira, Fernanda Priviero, Ana Delgado, Pengfei Dong, Valentina O. Mendoza, Linxia Gu, R. Clinton Webb, Kenia P. Nunes

**Affiliations:** 1 Laboratory of Vascular Biology, Department of Biomedical and Chemical Engineering and Sciences, Florida Institute of Technology, Melbourne, FL 32901, USA; 2 Cardiovascular Translational Research Center, Department of Cell Biology and Anatomy, University of South Carolina, Columbia, SC 29209, USA; 3 Laboratory of Bio-Mechanics, Department of Biomedical and Chemical Engineering and Sciences, Florida Institute of Technology, Melbourne, FL 32901, USA

**Keywords:** arterial stiffness, AFM, vascular contraction, collagen, elastin, sex differences

## Abstract

This study was designed to connect aortic stiffness to vascular contraction in young male and female Wistar rats. We hypothesized that female animals display reduced intrinsic media-layer stiffness, which associates with improved vascular function. Atomic force microscopy (AFM)-based nanoindentation analysis was used to derive stiffness (Young’s modulus) in biaxially (i.e., longitudinal and circumferential) unloaded aortic rings. Reactivity studies compatible with uniaxial loading (i.e., circumferential) were used to assess vascular responses to a selective $\alpha_{1}$ adrenergic receptor agonist in the presence or absence of extracellular calcium. Elastin and collagen levels were indirectly evaluated with fluorescence microscopy and a picrosirius red staining kit, respectively. We report that male and female Wistar rats display similar AFM-derived aortic media-layer stiffness, even though female animals withstand higher aortic intima-media thickness-to-diameter ratio than males. Female animals also present reduced phenylephrine-induced aortic force development in concentration-response and time-force curves. Specifically, we observed impaired force displacement in both parts of the contraction curve ($A_{phasic}$ and $A_{tonic}$) in experiments conducted with and without extracellular calcium. Additionally, collagen levels were lower in female animals without significant elastin content and fragmentation changes. In summary, sex-related functional differences in isolated aortas appear to be related to dissimilarities in the dynamics of vascular reactivity and extracellular matrix composition rather than a direct response to a shift in intrinsic media-layer stiffness.

## 1. Introduction

The leading cause of death globally, cardiovascular diseases (CVDs), dimorphically affects men and women [1,2]. Sex differences exist in the pathophysiology of CVDs, but the underlying mechanisms driving these dissimilarities are less clear. Pre-menopausal women are relatively protected against CVDs, whereas the incidence of CV events drastically increases after menopause [3], indicative of a critical role of sex hormones in this process. Likewise, pulse wave velocity (PWV) analysis revealed that pre-pubertal females have stiffer central arteries than age-matched males, and these differences disappear following puberty [4], a phenomenon that returns after menopause [5,6]. Arterial stiffening, the inability of large arteries to buffer pulsatile blood flow, independently predicts the development of CVDs [7,8,9]. Impaired aortic compliance modifies arterial pressure, leading to increased cardiac afterload and impaired perfusion of peripheral organs [10], while aging is a key risk factor for elevating arterial stiffness [11], the presence of comorbidities, such as diabetes [12,13], hypertension [14], and obesity [15], also precipitate the hardening of the aorta. Multiple mechanisms help explain sex-related differences in arterial stiffening [16]; however, it is less clear how sex as a variable influences arterial stiffness in healthy young subjects.

There are multiple approaches to quantifying arterial stiffness. The most widely used technique, PWV, non-invasively derives stiffness by calculating the transit time of the CV pulse [17]. However, PWV does not provide information about the mechanistic aspects (i.e., active and passive components) leading to variations in vessel stiffness, which can be altered by changes in endothelial function, vascular smooth muscle cells (VSMCs), extracellular matrix (ECM) [17], and perivascular adipose tissue (PVAT) [18]. Atomic force microscopy (AFM) provides nanoscale resolution of the biomechanical properties of soft biological tissues, including the vasculature [17,19]. AFM-based studies have successfully uncovered changes in arterial stiffness in response to hypertension [14,20], diabetes [21,22], and aging [14,23], but to our knowledge, it has not been used to map sex-related differences in media-layer stiffness under physiological conditions. Another useful technique to interrogate arterial stiffness is via functional studies incorporating uniaxial circumferential loading in a wire myograph. Large artery compliance is tightly regulated by VSM reactive properties, including contraction and relaxation [24], and there seems to be a linear relationship between stiffness and traction force development [25], which has been confirmed in diabetic coronary microvascular SMCs [21]. However, how AFM-based medial-layer stiffness results associate with force development in isolated aortas is unknown.

Accordingly, we designed this study to connect aortic stiffness to vascular contraction in young male and female Wistar rats. We hypothesized that female animals display reduced media-layer stiffness, which associates with improved vascular function. AFM-based nanoindentation analysis was used to measure stiffness (Young’s modulus) in biaxially unloaded (i.e., longitudinal and circumferential) aortic rings. Functional studies compatible with uniaxial loading (i.e., circumferential) were performed to assess vascular responses to phenylephrine, a commonly used $\alpha_{1}$ adrenergic agonist, in the presence or absence of extracellular calcium. The contribution of the ECM to this process was indirectly evaluated as a measure of elastin and collagen levels. Overall, it seems that, under the conditions evaluated in this study, sex-related functional differences in aortas are due to alterations in the dynamics of vascular reactivity and ECM composition rather than a direct response to a shift in intrinsic media-layer stiffness.

## 2. Results

Compared with male Wistar rats, female animals present lower body weight at 16 weeks (Table 1). The presence of diabetes and hypertension independently modify arterial stiffness; therefore, as shown in Table 1, we also confirmed that male and female animals are normoglycemic and normotensive.

### 2.1. AFM Stiffness Mapping of the Thoracic Aorta of Young Male and Female Wistar Rats

**Figure 1:**
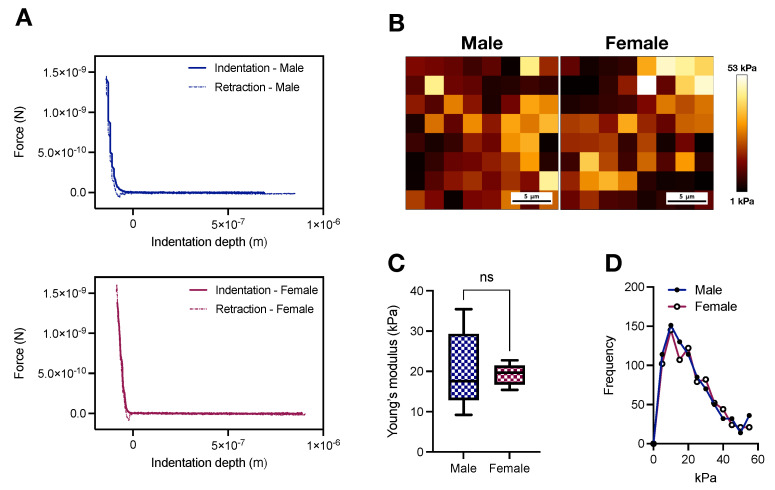
Male and female Wistar rats display similar atomic force microscopy-based media-layer stiffness at 16 weeks of age. (**A**) Representative force indentation curves and (**B**) AFM-based stiffness map for male and female samples. (**C**) Averaged Young’s modulus derived from the Hertz Model and (**D**) frequency of the measured stiffness. In (**C**), data are depicted as min to max, n = 5. ^ns.^ *p* > 0.05 using two-tailed Student’s *t*-test.

Arterial stiffening is intrinsically linked to the pathophysiology of CVDs [7,8,9], but how sex as a variable impacts aortic stiffness in physiological conditions is still elusive. We used an AFM-based tip size of 5 μm to cover an area of 25 μm^2^, determined in pilot experiments, to map stiffness in biaxially (i.e., longitudinal and circumferential) unloaded aortic rings (Figure 1A). Then, using the Hertz Model (Equation (1)), we derived the Young’s modulus in male and female samples (Figure 1B), which contrary to our initial hypothesis, revealed a similar average stiffness pattern in both sexes (20.4 ± 4.37 vs. 19.22 ± 1.21 kPa; male vs. female, *p* > 0.05, Figure 1C). Additionally, we did not observe substantial dissimilarities between sexes for the distribution of the measured stiffness (Figure 1D).

### 2.2. Histomorphometric Analysis of the Thoracic Aorta of Young Male and Female Wistar Rats

**Figure 2:**
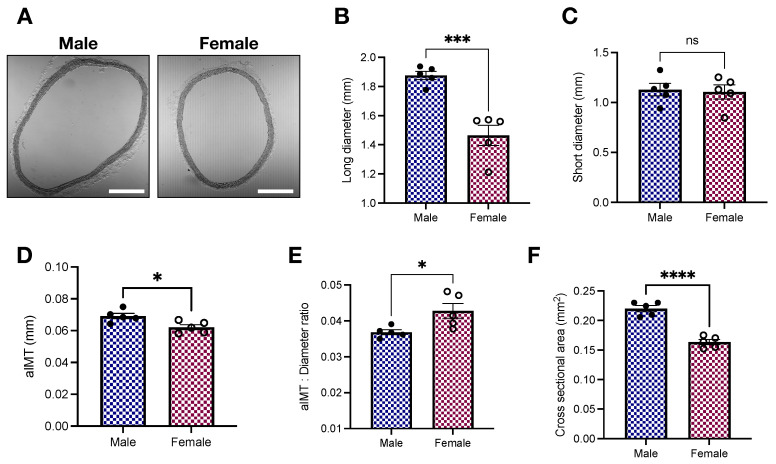
Young female Wistar rats display increased aIMT-to-diameter ratio. (**A**) Representative images, (**B**) long diameter, (**C**) short diameter, (**D**) aortic intima-media thickness (aIMT), (**E**) aIMT-to-diameter ratio, and (**F**) cross-sectional area. Data are presented as means ± SEM, n = 5. * *p* < 0.05, ***p < 0.001, **** *p* < 0.0001, and ^ns.^ *p* > 0.05 using two-tailed Student’s *t*-test. Scale bar: 500 μm.

Histological studies are key to determining the geometry of aortic rings [17,26] while providing indirect information about hemodynamics adaptations [27]. Our histomorphometric analysis showed that, in comparison with female samples, male aortic rings display increased lumen diameter (1.87 ± 0.02 vs. 1.46 ± 0.07 mm; *p* < 0.05, Figure 2B), aortic intima-media thickness (aIMT; 0.069 ± 0.002 vs. 0.062 ± 0.002 mm; *p* < 0.05, Figure 2D), and cross-sectional area (0.22 ± 0.005 vs. 0.16 ± 0.004 mm^2^; *p* < 0.05, Figure 2F). Consequently, the aIMT-to-diameter ratio is elevated in female animals (0.043 ± 0.002 vs. 0.037 ± 0.0007; *p* < 0.05, Figure 2E).

### 2.3. Vascular Responses to Phenylephrine in Young Male and Female Wistar Rats

**Figure 3:**
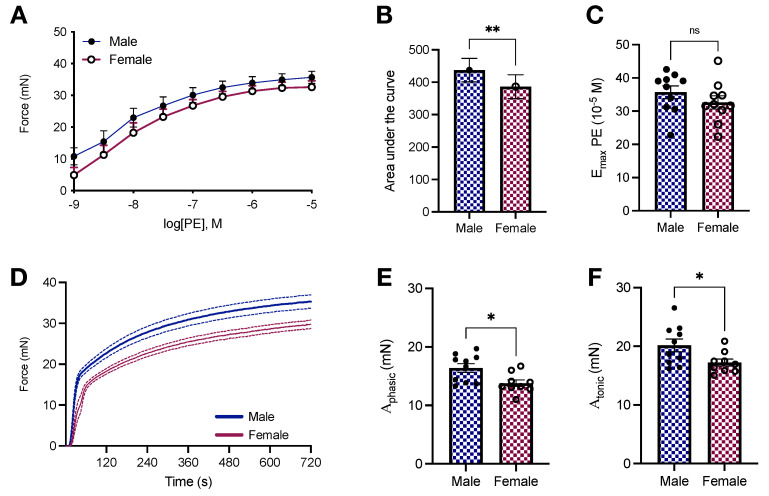
Female Wistar rats have reduced phenylephrine-induced force development in isolated aortas. (**A**) Concentration-response curve to phenylephrine (10^−9^–10^−4^ mol/L), (**B**) area under the curve, and (**C**) $E_{max}$. (**D**) Time-force curves and amplitude of the phasic (**E**) and tonic (**F**) components. All samples were stimulated with phenylephrine (10^−5^ mol/L). In D, data points were sample every second. The continuous line represents the mean, and the dashed lines depict SEM. Data are presented as means ± SEM, n = 9–10. * *p* < 0.05, ** *p* < 0.01, and ^ns.^ *p* > 0.05, using two-tailed Student’s *t*-test.

We previously showed that the aortas of male and female Sprague Dawley rats respond differently to phenylephrine stimulation [28], a feature also observed in Wistar Kyoto rats [29,30]. In this study, using Wistar rats, another outbred rat laboratory strain, we reproduced our findings and generated AFM-based stiffness data in the same animal, which allowed us to directly connect stiffness and contraction. As expected, there is a shift to the left in the concentration-response curve to phenylephrine in male animals (Figure 3A), which translates into a lower AUC in female aortic rings (386.4 ± 36.82 vs. 437.2 ± 36.15; *p* < 0.05, Figure 3B), but without significant changes in the maximum response elicited by the drug (32.61 ± 1.96 vs. 35.7 ± 1.91 mN; *p* > 0.05, Figure 3C). Then, we decided to investigate force-displacement in response to a single concentration of phenylephrine, which leads to the formation of a biphasic contraction curve. Female animals display reduced force development in both parts of the curve (Figure 3D). Specifically, we observed that the amplitude of the phasic ($A_{phasic}$; 13.79 ± 0.57 vs. 16.39 ± 0.75 mN; *p* < 0.05, Figure 3E) and tonic ($A_{tonic}$; 17.2 ± 0.6 vs. 20.15 ± 1.08 mN; *p* < 0.05, Figure 3F) parts of the contraction curve were significantly diminished in isolated female aortic rings.

**Figure 4:**
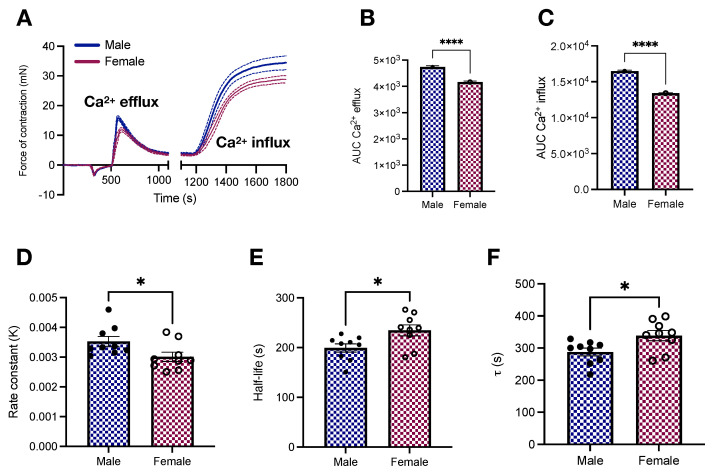
Vascular responses to calcium efflux and influx in male and female young Wistar rats. (**A**) Calcium protocol and (**B**,**C**) area under the curve. All samples were stimulated with phenylephrine (10^−5^ mol/L). In A, data points were sampled every second. The continuous line represents the mean, and the dashed lines depict SEM. The transient relaxation curves were fitted to a one-phase decay exponential model to reveal the rate constant κ (**D**), half-like (**E**), and τ (**F**). Data are presented as means ± SEM, n = 9–10. * *p* < 0.05 and **** *p* < 0.0001 using two-tailed Student’s *t*-test.

Afterward, we interrogated how sex affects vascular responses to phenylephrine-induced calcium dynamics. We found that female samples stimulated with phenylephrine have lower force development in response to calcium changes (Figure 4A). This led to a reduction in the AUC in female aortic rings in response to calcium efflux from the sarcoplasmic reticulum (4739 ± 46.67 vs. 4164 ± 43.18; *p* < 0.05, Figure 4B) and calcium influx via plasmalemmal channels (16,492 ± 114.5 vs. 13,408 ± 69.1; *p* < 0.05, Figure 4C). Female animals also displayed a tendency towards delayed time to peak (98.7 ± 11.95 vs. 93.33 ± 5.4 s; *p* < 0.05). Additionally, we further explored the dynamics of reactivity by fitting the relaxation component of the transient part of the calcium protocol to Equation (2). Curve fitting revealed a reduction in the rate constant κ (0.003 ± 0.0001 vs. 0.0035 ± 0.0001; *p* < 0.05, Figure 4D) coupled to an increase in the half-life (234.77 ± 11.03 vs. 199.42 ± 8.3 s; *p* < 0.05, Figure 4E) and time constant τ (338.71 ± 15.92 vs. 287.68 ± 11.98 s; *p* < 0.05, Figure 4F) in female aortic rings.

### 2.4. Elastin and Collagen Levels in the Aorta of Young Male and Female Wistar Rats

**Figure 5:**
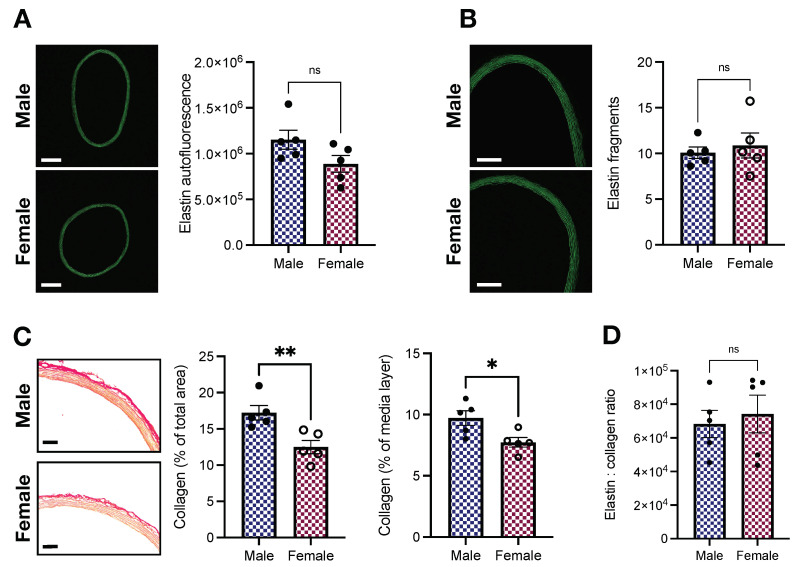
Female Wistar rats have lower levels of collagen without changes in elastin contents. (**A**) Elastin autofluorescence (arbitrary units), (**B**) elastin fragments (units), (**C**) collagen (picrosirius red), and (**D**) elastin:collagen ratio. Data are presented as means ± SEM, n = 5. * *p* < 0.05, ** *p* < 0.01, and ^ns.^ *p* > 0.05. Scale bars: 500 μm (**A**), 250 μm (**B**), and 100 μm (**C**).

The ECM, the non-cellular component of the vascular wall, contains fibrous proteins, including elastin and collagen [31]. Changes in ECM composition contribute to arterial stiffening [32]. Here, we indirectly assessed elastin and collagen levels with fluorescence microscopy and a picrosirius red staining kit, respectively. We found that, compared with female animals, male Wistar rats display a tendency towards higher levels of elastin (1,151,556.2 ± 104,838.1 vs. 888,221.4 ± 90,955.99 arbitrary units; *p* > 0.05, Figure 5A). No differences between sexes were observed for elastin fragmentation (10.08 ± 0.62 vs. 10.87 ± 1.36; male vs. female, *p* > 0.05, Figure 5B) nor the number of laminae (7.86 ± 0.35 vs. 7.8 ± 0.34; male vs. female, *p* > 0.05). On the other hand, we observed that male animals had enhanced collagen levels (Figure 5C). Differences were observed when we considered the total area of the vessel (17.22 ± 0.99 vs. 12.49 ± 0.92 %; male vs. female, *p* < 0.05, Figure 5C) and only the media-layer (9.72 ± 0.59 vs. 7.73 ± 0.39 %; male vs. female, *p* < 0.05, Figure 5C). However, the elastin-to-collagen ratio remained unaffected (68,269.07 ± 8141.59 vs. 74,188.51 ± 11,267.73; male vs. female, *p* > 0.05, Figure 5D).

## 3. Discussion

This study was designed to connect aortic stiffness to vascular contraction in male and female young Wistar rats, while sex as a variable affects arterial stiffness in response to aging and pathological conditions [16], there are still scant physiological data for females. In fact, a recent article highlighted that despite an increase in arterial stiffness research in the last decade, the inclusion of the variable sex reveals a dire lag in the field [33], contrasting with current guidelines for pre-clinical research. Here, we successfully used AFM-based nanoindentation to show, for the first time, that male and female young Wistar rats develop comparable intrinsic media-layer stiffness (Figure 1). As mentioned, AFM allows for nanoscale resolution of the biomechanical properties of biological samples [17,19]. By controlling the region used for AFM scanning, one derives definitive media-layer stiffness data, which are unloaded biaxially (i.e., longitudinally and circumferentially). These results can be used to understand the contributions of each vessel component to the overall observed in vivo material stiffness. This is important since targeting aortic stiffening resulting from the hardening of the muscle layer is different from tackling increased vessel stiffness in response to ECM remodeling, as well as endothelial dysfunction.

From our histomorphometric studies, we observed that female aortas withstand higher aIMT-to-diameter ratio than male vessels (Figure 2E). Young females (age: 8–17 years) are also exposed to increased loading in carotid arteries [27], highlighting the presence of this phenomenon in other vascular beds. Since media-layer stiffness is not significantly modified by sex at 16 weeks of age (Figure 1), such data indicate other mechanisms at play. For example, we found that female rings have less % collagen levels than males in the media-layer (Figure 5C). ECM changes, favoring low collagen levels, may be a compensatory mechanism to cope with increased aIMT-to-diameter ratio. Notably, enhanced collagen staining in males could also be responsible for inducing the heterogeneity (variation) of the stiffness measurement using AFM nanoindentation, even though it had no significant impact on the total average stiffness. We also did not observe significant changes in elastin autofluorescence or fragmentation between sexes (Figure 5A,B, respectively). It is well-accepted that ECM remodeling plays a causative role in arterial stiffness [32], but under certain conditions, arterial stiffening can still happen in direct response to changes in VSMCs [23], highlighting our findings’ significance. The concept of increased VSM stiffness as a key contributor to arterial stiffening has been demonstrated in hypertension [14,34] and aging [23], corroborating the idea that endothelial dysfunction and ECM remodeling are not the only players in these conditions. Here, we would like to appreciate that by presenting a similar stiffness pattern, young female animals might be at higher risk of developing stiffer arteries when faced with an adverse environment. Supporting this argument, even though young female animals, which are relatively protected from developing CVDs, are able to cope with the increased aIMT-to-diameter ratio, aging and diseases can still shift this balance. Females are at high risk of having increased arterial stiffness in response to weight gain [35]. Similarly, postmenopausal females lose protection against CVDs, displaying enhanced arterial stiffness [5,6]. However, whether the loss of female sex hormones is the only driver of this phenomenon is yet-to-be-determined.

We also performed functional studies compatible with uniaxial loading (i.e., circumferential) to evaluate the function of isolated aortas. This set of experiments allowed us to analyze vessel response, accounting for the cellular (ECs and VSMCs) and non-cellular components (ECM). Additionally, aortic rings loaded circumferentially are stronger than counterparts loaded perpendicular to ECM fiber direction, particularly because of the anisotropic behavior of blood vessels [26]. As expected, we found that female animals display reduced force development in response to adrenergic stimulation in concentration-response (Figure 3A,B) and time-force (Figure 3C,D) contraction curves. Similar results were obtained in the presence and absence of exogenous calcium (Figure 4A). Our data analysis of the transient component of the curve (relaxation) further revealed that female animals present a reduction in the rate constant κ (Figure 4D) coupled to an increase in the time constant τ (Figure 4F). Multiple mechanisms can account for reduced force-development in reactivity studies, while these results align with our previous study in male and female Sprague Dawley rats [28], by performing this set of experiments using another animal strain, our contributions are two-fold. We consolidate our findings in two of the most commonly used laboratory rat strains and we are able to directly relate arterial stiffness to vascular responses to phenylephrine in the same animal, avoiding inter-studies variations. Female animals have diminished levels of store-operated calcium channels, which translates into lower calcium influx and, consequently, diminished contraction [29]. Rho-kinase is yet another mechanism contributing to this process, mainly because estrogen inhibits this calcium sensitizer via activation of estrogen receptor [36]. Additionally, not only do female animals have high nitric oxide bioavailability [30,37], but also the levels of heat-shock protein 70, an emergent player in vascular biology, are reduced in female aortic rings [28], contributing to impaired force displacement. Together, these dynamic mechanisms coupled with morphometric differences (Figure 2) are likely the ones modulating the magnitude of contraction in female animals despite the comparable intrinsic media-layer stiffness between male and female rats.

We recognize that our methodology has limitations. First, biological samples might not be topographically and biomechanically homogeneous [19], while we were careful to collect aortic rings from the same thoracic region and to scan each sample in 2–4 different locations, variations could still be present in our analysis. Second, our functional studies only evaluated vascular responses to $\alpha_{1}$ adrenergic stimulation, which means that endothelial function was not directly assessed. Third, the contributions of PVAT to arterial stiffness were disregarded in this study, but recent evidence shows that vessel stiffness is lower when PVAT is present [18]. Notwithstanding these limitations, we firmly believe that our work narrows a fundamental knowledge gap: how sex as a variable affects intrinsic media-layer stiffness in young animals. Furthermore, this is the first work to attempt to connect information from AFM (nanoscale without biaxial load) and reactivity studies (ex vivo functional with uniaxial load) to understand vascular functional responses in both sexes.

## 4. Methods and Materials

### 4.1. Animals

Male and female Wistar rats (14 weeks) were acquired from Envigo, and housed under alternating light/dark cycles (12-h). Animals were subjected to a 2-week acclimation period in which they were fed standard chow diet and water *ad libitum*. Animals were euthanized by bilateral thoracotomy under isoflurane anesthesia (5% in 100% O_2_). Before euthanizing the animals, we determined non-fasted glucose levels in male and female rats using a commercially available blood glucose monitoring system. We also confirmed that animals displayed similar mean arterial pressure (MAP), as follows. A sterile catheter connected to a pressure transducer (model DT-100; Utah Medical Products) was inserted into the femoral artery (isoflurane; 2% in 100% O_2_), and blood pressure values were recorded for approximately 30 min.

### 4.2. Atomic Force Microscopy

Thoracic aorta sections (10 μm) from male and female animals were obtained on a cryostat. Sections were washed from optimal cutting temperature compound and AFM force-indentation measurements were performed with the JPK ForceRobot 300 AFM instrument. The scanning location was determined with the Zeiss Axio Observed inverted microscope. For the measurements, we used Bruker SAA/SPH probes consisting of silicon nitride tips (Tip radius: 5 μm, resonance frequency: 30 kHz) with a spring constant of 0.25 N/m. Since biological tissues are not topographically and biomechanically homogeneous, AFM scanning was performed at 2–4 independent locations per sample. For each scanned location, 64 (8 × 8 stiffness matrix) force curves were produced, covering an area of 25 μm^2^. The Young’s modulus (stiffness) was obtained by fitting the force-displacement curves to the Hertz Model [22,38,39], as follows.

(1) $$E=\;\frac{F\times 3\times (1\;-\;v^{2})}{4\sqrt{R}{\;\delta }^{3/2}}$$

where, *E* is the Young’s modulus, *v* is the Poisson’s ratio, *R* is the tip radius, and δ is the indentation depth. Force curves that did not display an appropriate indentation and retraction pattern were discarded from the analysis. The reported stiffness was generated by first averaging the Young’s modulus resultant from the viable force displacement curves of each location, and then, averaging the independent measurements for each animal. The JPK PD software (Bruker Nano Inc., Santa Barbara, CA, USA; version 6.1.166) was used to fit the curves.

### 4.3. Histomorphometric Analysis

Aortic sections (10 μm) were washed in PBS and mounted onto glass slides. Digital images were immediately obtained in a Nikon Eclipse Ti2 inverted microscope (4× magnification). The ImageJ software (NIH, Bethesda, MD, USA) was used to determine the diameter (short and long axis; mm), aIMT (mm; average of three measurements), and cross-sectional area (mm^2^).

### 4.4. Functional Studies

Functional studies were performed in a DMT620M multi-wire myograph system (Danish MyoTechnology, Aarhus, Denmark) [28,40,41,42]. Briefly, 2 mm endothelium-intact rings were obtained from the thoracic aorta, and mounted with a physiological preload of 15 mN/mm. Since PVAT modifies aortic stiffness [18], studies were conducted in PVAT-denuded vessels. Each myograph chamber was filled with physiological salt solution (PSS; mmol/L: 130 NaCl, 4.7 KCl, 1.18 KH_2_PO_4_, 1.18 MgSO_4_ * 7H_2_O, 14.9 NaHCO_3_, 5.6 Dextrose, 1.56 CaCl_2_ * H_2_O, 0.026 EDTA), which was gassed with carbogen (95% CO_2_/ 5% O_2_, pH: 7.4) and kept at 37 ${}^{\circ }$C. Samples were allowed to equilibrate for approximately 1 h. To test for the viability of the rings, we used a high K^+^ solution (120 mmol/L for 10 min) modified from PSS. Viable rings (force development > 50% of preload) were washed and allowed to return to baseline. Note that we did not observe differences between sexes for the maximum contraction tone elicited by KCl (36.25 ± 2.15 vs. 36.99 ± 1.55 mN; *p* > 0.05, male vs. female). Then, rings were stimulated with a single concentration of phenylephrine (10^−5^ mol/L; time-force curve) for 12 min. Samples were washed and allowed to return to baseline. Afterwards, we obtained a concentration-response curve to phenylephrine (10^−9^–10^−4^ mol/L). In another set of experiments, following the viability test, we assessed vascular responses to calcium [28,42,43]. Briefly, the PSS was replaced with calcium free PSS for 3 min. A single concentration of phenylephrine (10^−5^ mol/L) was used to stimulate calcium release from internal stores. Contraction was recorded for 10 min. Then, we restored the calcium concentration to the PSS (1.56 CaCl_2_ * H_2_O mol/L) and observed force displacement in response to calcium influx for 15 min.

The $E_{max}$ of the concentration-response curves was determined at 10^−5^ mol/L. In the time-force and calcium protocol, data points were sampled every second and are reported as absolute force development from basal levels. The amplitude of the phasic ($A_{phasic}$) and tonic ($A_{tonic}$) parts of the time-force curves was calculated following the protocol outlined by our research group [28]. The trapezoidal method was used to calculate the total area under the curve (AUC) in all contraction curves. The transient relaxation curves in the calcium protocol were fitted to a one-phase decay exponential model as follows.

(2) $$F=F_{0}\times exp^{\left(-\kappa \times T\right)}$$

where, *F* is force, $F_{0}$ is *F* when time (*T*) is equal to 0, and κ is the rate constant. We set plateau to be a constant equal to 0. From the model we also extracted the time constant τ, and the half-life, which was computed as $\frac{ln(2)}{\kappa }$.

### 4.5. Elastin Characterization

Aortic rings (10 μm) from both sexes were excited at 488 nm with equal exposure time [44]. Autofluorescence values are reported as arbitrary units, which were computed with the ImageJ software in images without the background (4× magnification). Number of laminae was manually evaluated, and is reported as the average of independent measurements across three regions (top, middle, bottom) of the image. Number of elastin fragments were determined with the threshold function in the ImageJ software (10× magnification) [44]. Representative images were modified for brightness and contrast using the same parameters.

### 4.6. Collagen Detection

Collagen levels were assessed in aortic rings (10 μm) with a commercially available kit (Abcam, ab150681), following the protocol provided by the manufacturer. Images were acquired after 24 h in a Zeiss Model AxioSkop-2 MOT microscope (10× magnification). Samples were analyzed with the ImageJ software to determine collagen deposition (% of positive area; red staining) in the media-layer and total vessel (media-layer + adventitia). Representative images were modified for brightness and contrast using the same parameters.

### 4.7. Statistical Analysis

The software GraphPad Prism (version 9.0) was used to create the graphs, which are depicted as means ± SEM, unless stated otherwise, and to compute significance of differences between male and female samples. The statistical test applied was the two-tailed Student’s *t*-test (unpaired), and statistical significance was considered for values of p≤0.05. Outliers were detected with the two-sided Grubbs test.

## 5. Conclusions

It seems that, under the conditions evaluated in this study, the observed sex-related differences in aortic function are due to alterations in the dynamics of vascular reactivity and ECM composition rather than a direct response to a shift in intrinsic media-layer stiffness. Broadly speaking, our work provides a critical piece of information (i.e., female vs. male baseline stiffness data) for researchers modeling arterial stiffness in non-pathological (e.g., aging) and pathological (e.g., diabetes, hypertension) conditions.

## Figures and Tables

**Table 1:** Animal profile. Data are presented as means ± SEM, n = 5. * *p* < 0.05 and ^ns.^ *p* > 0.05 using two-tailed Student’s *t*-test.

Group	Body Weight (g)	Glucose Levels (mg/dl)	Mean Arterial Pressure (mmHg)
Male	495.4 ± 9.74	154.2 ± 18.02	87.6 ± 3.93
Female	228.4 ± 5.32 ${}^{*}$	181.4 ± 8.99${}^{\;\mathrm{ns}.}$	89.8 ± 2.95${}^{\;\mathrm{ns}.}$

## Data Availability

The data supporting the findings of this study are available from the corresponding authors upon reasonable request.
